# CARD*Shark: automated prioritization of literature curation for the Comprehensive Antibiotic Resistance Database

**DOI:** 10.1093/database/baad023

**Published:** 2023-04-20

**Authors:** Arman Edalatmand, Andrew G McArthur

**Affiliations:** David Braley Centre for Antibiotic Discovery, McMaster University, 1280 Main Street West, Hamilton, ON L8S 4L8, Canada; Michael G. DeGroote Institute for Infectious Disease Research, McMaster University, 1280 Main Street West, Hamilton, ON L8S 4L8, Canada; Department of Biochemistry and Biomedical Sciences, McMaster University, 1280 Main Street West, Hamilton, ON L8S 4L8, Canada; David Braley Centre for Antibiotic Discovery, McMaster University, 1280 Main Street West, Hamilton, ON L8S 4L8, Canada; Michael G. DeGroote Institute for Infectious Disease Research, McMaster University, 1280 Main Street West, Hamilton, ON L8S 4L8, Canada; Department of Biochemistry and Biomedical Sciences, McMaster University, 1280 Main Street West, Hamilton, ON L8S 4L8, Canada

## Abstract

Scientific literature is published at a rate that makes manual data extraction a highly time-consuming task. The Comprehensive Antibiotic Resistance Database (CARD) utilizes literature to curate information on antimicrobial resistance genes and to enable time-efficient triage of publications we have developed a classification algorithm for identifying publications describing first reports of new resistance genes. Trained on publications contained in the CARD, CARD*Shark downloads, processes and identifies publications recently added to PubMed that should be reviewed by biocurators. With CARD*Shark, we can minimize the monthly scope of articles a biocurator reviews from hundreds of articles to a few dozen, drastically improving the speed of curation while ensuring no relevant publications are overlooked.

**Database URL** http://card.mcmaster.ca

## Introduction

Antimicrobial resistance (AMR) is a health-care crisis estimated to cause the death of 10 million individuals and cost $100 trillion worldwide by 2050 (1). Antimicrobial misuse in both clinical and agricultural settings has undermined the effectiveness of molecules responsible for an otherwise miracle of modern medicine (2), i.e. our ability to control infection by bacterial pathogens since the early 20th century. Yet even as bacteria evolve mechanisms to resist current antimicrobials, the discovery pipeline for new candidate molecules has become moribund (3, 4). With this dire background, phenotypic and genotypic surveillance of AMR is increasingly important at regional, national and international levels to assess risk and inform policy, while new machine learning approaches hold promise for personalized medicine based on pathogen genome sequencing to guide treatment of individuals while favoring stewardship of antimicrobials for the broader community (5–8).

Understanding the mechanisms underlying AMR is of great importance for the targeted treatment of bacterial infections and for community risk assessment. With an understanding of the suite of AMR genes (ARGs) found in a pathogen together with the antibiotics they confer resistance toward, we come closer to predicting the phenotypic antibiogram of a pathogen (9). As such, sequencing of pathogen genomes from individual infections, among communities, and in different environments is in increasing use in research, public health and industry. With this increased effort in sequencing of bacterial pathogens comes the need for accurate annotation of ARGs within these genome sequences and an increasing number of tools and databases exist for this annotation effort. While many are specialized to specific pathogens, drug classes or mechanisms of resistance, several databases and associated tools attempt to annotate the entire catalog of known ARGs for genome assemblies or metagenomic reads, foremost among these being the Comprehensive Antibiotic Resistance Database (CARD) (10), ResFinder (11) and the National Center for Biotechnology Information Pathogen Detection Reference Gene Catalog (12). Yet, AMR is a unique curation challenge as the antimicrobial resistome spans numerous environments, and the misuse of antimicrobials can create selective pressure upon new targets, leading to new resistance ARGs and mechanisms. Furthermore, ARGs are often associated with mobile genetic elements such as plasmids, allowing ARGs emerging in environmental settings to move into agricultural settings and follow the farm-to-fork route to clinical settings. Novel ARGs are quickly being discovered with the expansion of next-generation sequencing efforts, and it has become difficult for teams of biocurators to efficiently identify the subset of literature discussing novel ARGs or mutations within the corpus of the AMR literature (∼8000 new publications per year).

CARD is an ontological-driven database of ARGs and individual mutations conferring resistance in a broad range of bacterial species, including those found in clinical, agricultural and environmental settings (10). Powered by the Antibiotic Resistance Ontology (ARO), CARD seeks to catalog all known ARGs, their products and associated phenotypes within both an ontological context (i.e. connecting ARGs to antibiotics, AMR mechanisms, and evolution from drug targets) and bioinformatic context (i.e. collating reference sequences and mapped mutations in the context of bioinformatic parameters for their accurate prediction in new genomic data). Upon this knowledge base, CARD layers software tools for genome or metagenome annotation, design of bait capture platforms, and annotation of >100 000 genomes, plasmids or shotgun assemblies to provide comprehensive resistome, variants, and prevalence information. Yet, at its base CARD relies heavily on manual, expert curation of ARGs (10, 13). In particular, curators seek to ensure CARD and the ARO have information on all ARGs upon their first description and that ‘to be included in CARD an AMR determinant must be described in a peer-reviewed scientific publication, with its DNA sequence available in GenBank, including clear experimental evidence of elevated minimum inhibitory concentration (MIC) over controls’ (10). Given the high pace of the discovery of new ARGs, manual triage of the scientific literature proved inefficient, and the CARD team developed word-association scoring matrices to prioritize publications in PubMed for manual investigation by curators (10, 13). However, this method proved to have several limitations, outlined below, and here we report the development of supervised learning methods for the identification and prioritization of scientific publications containing newly described ARGs for curation into CARD. This work is informative for the development of automated or algorithm-assisted curation of the scientific literature and provides details on CARD’s approach for ensuring comprehensive curation of ARGs for annotation of newly sequenced genomes or metagenomes.

## Materials and methods

### Prioritization via word-association scoring matrices (CARD*Shark 2)

The CARD team initially attempted the use of word-association scoring matrices to prioritize papers in PubMed. After two rounds of development, the Python-based CARD*Shark 2 algorithm guided curators to relevant papers using an algorithm that scored papers based on their abstract similarity to papers previously curated in CARD for ARGs. For example, for macrolide antibiotics, using the relative frequencies of each word in the abstracts of all papers associated with macrolides in CARD, CARD*Shark 2 calculates a score for each macrolide-associated paper by taking the sum of relative frequencies of all abstract words. Based on the score, it assigned new papers in PubMed to a low- or high-level group that dictates the papers’ relevance to curators if the score falls below or above a cut-off, respectively. For our analysis, papers assigned to the high-level group are considered positive predictions, and low-level papers are considered negative predictions. Although the algorithm provided curators with a reasonable set of papers to triage, there were a few key limitations. First, CARD*Shark 2 pulled papers from PubMed on a monthly basis using only drug class terms found in CARD’s ARO as query terms, ignoring words for individual antibiotics or ARGs and thus potentially missing important papers when an abstract does not discuss a drug class directly. In addition, papers in PubMed can include multiple drug class terms and are thus scored differently by each drug class–specific scoring matrix. Such papers could be reported under multiple drug class search results, creating redundancy and inefficiency for curators, but also inconsistency as papers could appear low level in one drug class group but high level in another. Finally, every paper CARD*Shark 2 examined was considered a positive result even if the initial PubMed query result included non-AMR papers, yet the scoring was often not discriminatory and forced a manual review of all papers by curators. With these flaws in mind, we were interested in implementing a machine learning version of CARD*Shark that reviewed all recently added PubMed articles monthly and identified all relevant publications with high recall, while not overburdening the curator with too many false positives, i.e. low precision.

### Paper retrieval, preprocessing and feature extraction for supervised learning

The type of problem CARD*Shark 2 aims to solve is one of binary classification: given an input of publications newly added to PubMed, we wish to assign a pass/fail on whether they contain valuable new information for curation into CARD. Many machine learning algorithms can solve binary classification problems and we examined these as possible improvements upon CARD*Shark 2. Machine learning classification algorithms fall into two broad categories: supervised learning and unsupervised learning (14). Unsupervised learning methods use clustering methods on unlabeled data to identify unknown groups of data (14), while supervised algorithms require two steps: a training step and a prediction or testing step. For supervised algorithms, the training step involves using a processed, pre-labeled set of data that can be fed to the algorithm to generate a model. This model is then used to predict results for performance analysis and subsequent real-world application.

For the classification of the scientific literature, we examined supervised classification algorithms including logistic regression, naive Bayes, random forest, extreme gradient boosting and support vector machines to determine the best-performing approach to guide the curation of papers into CARD. To create prediction models for classification tasks, we used PubMed’s Entrez application programming interface (API) to retrieve abstracts and metadata from PubMed’s database (15) as a step toward creating a set of features for training models. The retrieved papers were then preprocessed using the Natural Language Toolkit (NLTK) and the regular expression package in python (16) to help normalize and reduce redundancy within the text (17). Preprocessing is an important step to help improve model performance by removing any non-essential terms from the text, removing punctuation, converting terms to their base form and removing digits (17). Using regular expressions, we first removed punctuation from abstracts. Then, after splitting abstracts into individual words, stopwords provided by the NLTK package were removed along with digits. A Porter stemmer then converted each remaining word to its base form (18), and collectively these preprocessing steps resulted in abstracts containing a series of normalized words.

Once preprocessed, the procedure of converting data into usable vectors for machine learning is called feature extraction and aims to reduce the dimensionality of data for algorithmic use. The two major types of dimensionality reduction that can be applied to text include bag-of-words (BOW) and term frequency–inverse document frequency (TF–IDF) (19, 20). BOW, like its name suggests, involves counting the number of occurrences of a word in a document. TF–IDF can be considered a more sophisticated version of BOW as it takes the frequency of words appearing in a text and multiplies it by the logarithm of the inverse fraction of documents containing the word (19). TF–IDF helps classifiers ignore words that appear too commonly, giving more weight to other words more relevant to the information extraction task at hand. To assess the relative strengths of each approach, the normalized abstracts were converted into usable vectors following three approaches using scikit-learn (21): TF–IDF on bigrams and trigrams, BOW, and TF–IDF on individual words. Each method was applied separately to produce three separate training/testing sets for evaluation by each of the different supervised learning approaches.

### Model training and cross-validation

To determine the best classifier and using scikit-learn (21), we trained and tested logistic regression, naive Bayes, random forest, extreme gradient boosting and support vector machines through 5-fold stratified cross-validation for each set of normalized abstracts. We trained/tested models on a set of 9710 papers, of which 1886 papers were previously curated into CARD within ‘AMR Gene Family’ tagged ontology terms as our positive set. The remaining 7824 papers acted as our negative set, i.e. low-scoring predictions obtained from CARD*Shark 2 but not added to CARD by human curators. Of the total set of papers, 75% was used for initial model training and testing through cross-validation, with the remaining 25% of papers used for a final holdout evaluation. After 5-fold cross-validation, we calculated precision (i.e. the proportion of papers classified as curation-worthy that were in the 1886 paper positive set), recall (i.e. the proportion of the 1886 paper positive set classified as curation-worthy) and the *F*_1_ statistic (i.e. the harmonic mean of precision and recall). For comparison, we also scored the 9710 papers using CARD*Shark 2 with 5-fold stratified cross-validation.

### Human validation

Papers for September and November 2019 were obtained from PubMed using the MeSH ‘[Date—Create]’ query via the Entrez API (i.e. ‘start date’ [Date - Create]: ‘end date’ [Date - Create]). We processed these papers, as outlined earlier, and classified them using the models generated from cross-validation. For independent human validation of the classifiers, 100 papers were randomly selected from the September logistic regression predictions, with an even 50/50 split between classification results (i.e. 50% predicted positive and 50% predicted negative), while for the November set, a total of 330 papers were selected for validation (half provided by selecting an even split of predictions from naive Bayes and the other half from an even split of predictions from random forest). Subsequently, a group of 11 experienced CARD curators reviewed each paper by answering a series of three questions to generate binary labels for each paper, with two individuals reviewing each paper. The subset of algorithms examined during human validation was based on the best-performing approaches during initial investigations, instead of all possible algorithms, to reduce the overall effort required by our human curators. The three questions asked of the human curators were the following: ‘Is there an AMR gene described in the abstract’, ‘Is there an elevated MIC anywhere in the paper’ and ‘Would you curate the gene into CARD based solely on the abstract?’ If conflicting labels arose, the paper was not included in the final validation results; otherwise, the human curator results were compared to the supervised model predictions as an independent assessment of performance.

## Results and discussion

**Figure 1.**
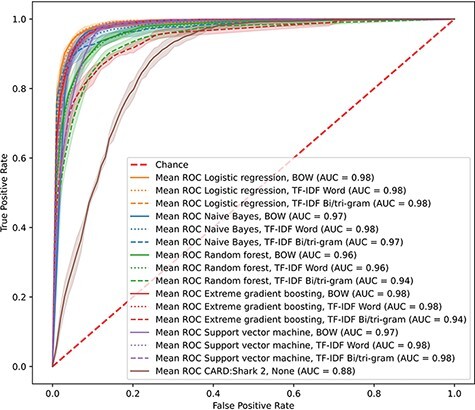
CARD*Shark 2 and supervised learning cross-validation ROC curves. Results from 5-fold cross-validation of several supervised learning models using three feature extraction methods, plus CARD*Shark 2. Results from all five cross-validation tests were averaged to produce a single curve. Shadows around each line are ±1SD from the mean. For CARD*Shark 2, high-level predictions are considered positive predictions, and low-level predictions are considered negative predictions.

With the overwhelming number of papers added every month to PubMed, it is difficult for curators to triage the whole scope of domain-specific literature. CARD had previously developed CARD*Shark 2 to address the issue of reducing the number of papers individual curators needed to review to identify novel ARGs. However, there were limitations with this word-association scoring algorithm in both the scope of papers it scored and the grouping of papers. To improve upon CARD*Shark 2 while expanding the scope of papers examined, we created and tested a set of supervised learning models that would predict whether a paper contained information appropriate for curation into CARD. To identify the best-performing model, we evaluated five supervised learning methods through cross-validation. All the supervised learning models achieved receiver operating characteristic (ROC) area under the curves of >0.94, while CARD*Shark 2 obtained an area under the curve of 0.88 (Figure 1). Similarly, the precision and *F*_1_ results of CARD*Shark 2 underperformed in comparison to most supervised learning models (Table 1), although not surprisingly CARD*Shark 2 was able to recall 97% of papers already curated into CARD while suffering from low precision (i.e. also predicting many unworthy papers as curation-worthy). Overall, results from cross-validation of the supervised learning models showed that these methods can achieve a better performance compared to the current CARD*Shark 2 algorithm. However, solely based on cross-validation, it was difficult to discern the best-performing supervised learning model.

**Table 1.** CARD*Shark 2 and supervised learning cross-validation precision-recall statistics

Model name	Feature extraction method	Precision	Recall	*F*_1_
Logistic regression	TF–IDF bi/trigram	0.96	0.41	0.58
	TF–IDF word	0.92	0.81	0.86
	BOW	0.89	0.88	0.89
Naive Bayes	TF–IDF bi/trigram	1.00	0.26	0.41
	TF–IDF word	0.99	0.36	0.52
	BOW	0.74	0.96	0.83
Random forest	TF–IDF bi/trigram	0.83	0.60	0.70
	TF–IDF word	0.89	0.66	0.76
	BOW	0.90	0.66	0.76
Extreme gradient boosting	TF–IDF bi/trigram	0.84	0.65	0.74
	TF–IDF word	0.89	0.81	0.85
	BOW	0.89	0.81	0.85
Support vector machine	TF–IDF bi/trigram	-	0.00	0.00
	TF–IDF word	-	0.00	0.00
	BOW	0.96	0.15	0.26
CARD*Shark 2		0.38	0.97	0.54

Results from 5-fold cross-validation of several supervised learning models using three feature extraction methods, plus CARD*Shark 2. Underlined numbers represent the best-performing method for each category. For CARD*Shark 2, high-level predictions are considered positive predictions, and low-level predictions are considered negative predictions.

**Table 2.** Model validation results and predictions for November and September 2019 papers

Model name	Feature extraction method	FN	FP	TN	TP	Precision	Recall	*F*_1_	Negative predictions	Positive predictions
Logistic regression	TF–IDF bi/trigram	7	6	397	1	0.14	0.13	0.13	241 618	187
	TF–IDF word	4	42	361	4	0.09	0.50	0.15	240 773	1032
	BOW	4	96	307	4	0.04	0.50	0.07	237 149	4.656
Naive Bayes	TF–IDF bi/trigram	8	0	403	0		0.00	0.00	241 796	9
	TF–IDF word	8	0	403	0		0.00	0.00	241 788	17
	BOW	1	92	311	7	0.07	0.88	0.13	237 126	4679
Random forest	TF–IDF bi/trigram	3	33	370	5	0.13	0.63	0.22	240 570	1235
	TF–IDF Word	7	30	373	1	0.03	0.13	0.05	240 163	1642
	BOW	5	38	365	3	0.07	0.38	0.12	240 023	1782
Extreme gradient boosting	TF–IDF bi/trigram	5	28	375	3	0.10	0.38	0.15	240 882	923
	TF–IDF word	4	31	372	4	0.11	0.50	0.19	240 165	1640
	BOW	6	27	376	2	0.07	0.25	0.11	240 290	1515
Support vector machine	TF–IDF bi/trigram	8	0	403	0		0.00	0.00	241 805	0
	TF–IDF word	8	0	403	0		0.00	0.00	241 805	0
	BOW	7	2	401	1	0.33	0.13	0.18	241 742	63
CARD*Shark 2		0	165	238	8	0.05	1	0.09	200 906	40 899

TP, TN, FP, FN, precision, recall and *F*_1_ values represent validation of a random subset of papers by human curators, with underlined numbers representing the best-performing method for precision, recall and *F*_1_. Negative predictions represent the number of papers that CARD curators would ignore, while positive predictions are the number of papers requiring review by CARD curators for possible new additions to CARD. For CARD*Shark 2, high-level predictions are considered positive predictions, and low-level predictions are considered negative predictions. TP, true positive; TN, true negative; FP, false positive; FN, false negative.

To gain a better understanding of each model’s performance in a real-world application, each model made predictions on all papers for September and November 2019 (Table 2). A random subset of these papers was used for independent human validation. The main goal of these models was to achieve a high recall value as missing a novel ARG because of a poorly performing model is detrimental to CARD’s overall objectives. The secondary objective of these models was a high precision to reduce the number of papers curators must review. Based on human validation, all the supervised learning models resulted in low precision values of <34%, while naive Bayes obtained the highest recall of 88% (Table 2). Despite high recall by naive Bayes, low precision is undesirable as it would result in too many papers for manual review. If we instead focus on models with a good balance between precision and recall via *F*_1_ values, we find logistic regression, naive Bayes, random forest and extreme gradient boosting each have one model that performs with the highest *F*_1_ score, with random forest on TF–IDF on bi/trigrams having the best overall performance (Table 1). CARD*Shark 2 performs with the best recall (100%) at the cost of having the third lowest precision. The impact of CARD*Shark 2’s low precision can be seen in the 40 899 papers it flags for curator review, more than an order of magnitude higher than any of the supervised learning methods. Future improvements to precision and recall may require the use of an ensemble of the best-performing models. Notably, only 430 papers were selected for human validation out of a set of >200 000 papers, and as such, we cannot definitively conclude that one model is better than another until more papers are evaluated. A more significant issue faced by both CARD*Shark 2 and the supervised learning models is that we do not know the extent of relevant papers being ignored as CARD does not keep a record of negative curations. Moving forward, it would be advisable to mix a subsample of negative predictions into the curation set to evaluate ignored essential papers.

**Figure 2.**
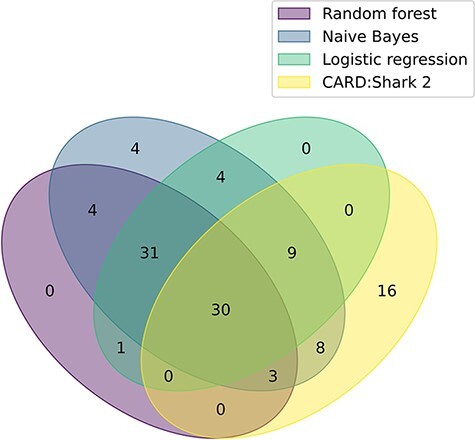
A Venn diagram illustrating the overlap of each model’s positive paper predictions that were ultimately curated into CARD. The plot based on data from Table 3. For CARD*Shark 2, both high- and low-level predictions are included.

Continued evaluation of logistic regression, random forest and naive Bayes is being performed through monthly paper predictions that are assessed by CARD’s team of curators. Additionally, a retrospective analysis of each of the models was conducted by predicting papers for the majority of months CARD*Shark 2 has been running (1 July 2017–1 November 2020). During this time, CARD*Shark 2 flagged 22 196 unique papers, 66 of which were added to CARD by the curators (Table 3). The benefit of the expanded scope of the supervised learning models can be seen in Figure 2, where 44 papers were successfully identified by the models but never flagged by CARD*Shark 2. At the same time, CARD*Shark 2 was able to identify 16 papers the supervised learning models missed (Figure 2). These results indicate that a combination of CARD*Shark 2 and the supervised learning models may be necessary to identify papers for curation into CARD.

**Table 3.** Retrospective predictions against papers added to PubMed between July 2017 and December 2020

Model name	Papers examined	Positive predictions	Added to CARD
Logistic regression	3 955 928	30 049	75
Naive Bayes		75 843	93
Random forest		17 318	69
CARD*Shark 2	22 196	H: 10 676;L: 11 520	H: 58; L: 8

CARD*Shark 2 categorizes its predictions into an L or H level. L, low; H, high.

## Conclusion

Overall, we have found that supervised learning applications to rapidly triage thousands of publications can viably reduce the burden associated with curating data. However, due to the limited scope associated with CARD’s curation goal (i.e. identifying new ARGs only), models perform with poor precision but high recall. To compensate for this precision, a combination of CARD*Shark 2 and the supervised learning models will be incorporated into CARD by ranking publications based on model agreement to maintain high recall while prioritizing high-value publications (i.e. publications with the highest model agreement are reviewed first). As such, a computer-guided curation paradigm that centers ultimately on expert, human biocuration allows CARD to provide comprehensive, high-value, trustworthy data for genomic surveillance of AMR.

## Data Availability

Software for CARD*Shark is available at https://github.com/edalatma/card_shark_3.
